# Priming With Intermittent Theta Burst Transcranial Magnetic Stimulation Promotes Spinal Plasticity Induced by Peripheral Patterned Electrical Stimulation

**DOI:** 10.3389/fnins.2018.00508

**Published:** 2018-07-24

**Authors:** Tomofumi Yamaguchi, Toshiyuki Fujiwara, Su-Chuan Lin, Yoko Takahashi, Kozo Hatori, Meigen Liu, Ying-Zu Huang

**Affiliations:** ^1^Department of Physical Therapy, Yamagata Prefectural University of Health Sciences, Yamagata, Japan; ^2^Department of Rehabilitation Medicine, Keio University School of Medicine, Keio University, Tokyo, Japan; ^3^Postdoctoral Fellow for Research Abroad (JSPS), Tokyo, Japan; ^4^Department of Neuroscience, University of Copenhagen, Copenhagen, Denmark; ^5^Department of Rehabilitation Medicine, Juntendo University Graduate School of Medicine, Juntendo University, Tokyo, Japan; ^6^Neuroscience Research Center and Department of Neurology, Chang Gung Memorial Hospital and Chang Gung University College of Medicine, Taoyuan, Taiwan; ^7^Institute of Cognitive Neuroscience, National Central University, Taoyuan, Taiwan

**Keywords:** H-reflex, disynaptic reciprocal inhibition, presynaptic inhibition, spinal plasticity, non-invasive brain stimulation

## Abstract

This study explored the effect of corticospinal activity on spinal plasticity by examining the interactions between intermittent theta burst transcranial magnetic stimulation (iTBS) of the motor cortex and peripheral patterned electrical stimulation (PES) of the common peroneal nerve (CPN). Healthy volunteers (*n* = 10) received iTBS to the tibialis anterior (TA) muscle zone of the motor cortex and PES of the CPN in three separate sessions: (1) iTBS-before-PES, (2) iTBS-after-PES, and (3) sham iTBS-before-PES. The PES protocol used 10 100-Hz pulses every 2 s for 20 min. Reciprocal inhibition (RI) from the TA to soleus muscle and motor cortical excitability of the TA and soleus muscles were assessed at baseline, before PES, and 0, 15, 30, and 45 min after PES. When compared to the other protocols, iTBS-before-PES significantly increased changes in disynaptic RI for 15 min and altered long-loop presynaptic inhibition immediately after PES. Moreover, the iTBS-induced cortical excitability changes in the TA before PES were correlated with the enhancement of disynaptic RI immediately after PES. These results demonstrate that spinal plasticity can be modified by altering cortical excitability. This study provides insight into the interactions between modulation of corticospinal excitability and spinal RI, which may help in developing new rehabilitation strategies.

## Introduction

Synaptic plasticity in the spinal cord plays an important role in functional recovery of the lower extremities and in recovery of walking function after spinal cord injury (SCI) and stroke (Wolpaw and Tennissen, 2001; Wolpaw, 2007; Wang et al., 2012; Howlett et al., 2015; Yamaguchi et al., 2016). Therefore, a rehabilitative strategy for enhancing spinal plasticity could be beneficial for patients with central nervous system lesions.

One strategy for the enhancement of spinal plasticity is peripheral patterned electrical stimulation (PES), which enhances spinal reciprocal inhibition (RI) (Perez et al., 2003). However, in patients with incomplete SCI the plasticity induced by PES is only observed immediately after stimulation (Yamaguchi et al., 2016), and in healthy individuals it only lasts 10 min at most (Perez et al., 2003; Fujiwara et al., 2011; Yamaguchi et al., 2016; Takahashi et al., 2017). The short duration of the effects of PES limits its clinical applicability for rehabilitation. Therefore, new strategies to enhance spinal plasticity are needed.

There are indications that supraspinal modulation may strengthen the plastic changes in spinal circuits that are induced by PES (Chen et al., 2006; Fujiwara et al., 2011; Yamaguchi et al., 2013, 2016; Takahashi et al., 2017). Transcranial direct current stimulation (tDCS) is a non-invasive brain stimulation technique that can alter motor cortex excitability (Nitsche and Paulus, 2000). The spinal plasticity induced by PES can be modulated by anodal and cathodal tDCS (Fujiwara et al., 2011). Yamaguchi et al. (2016) reported that anodal tDCS of the motor cortex extended subsequent PES-induced plastic changes in spinal RI to at least 20 min, even in patients with incomplete SCI. These results suggest that combining anodal tDCS and PES may be a useful strategy for enhancing spinal plasticity. However, the cited studies did not examine changes in motor cortical excitability, so the relationship between motor cortical excitability and PES-induced spinal plasticity remains unclear.

The time sequence of cortical stimulation and sensory inputs may also be important. Many studies have reported effects of stimulus timing on the efficacy of interventions capable of inducing plasticity (Iyer et al., 2003; Ridding and Ziemann, 2010; Müller-Dahlhaus and Ziemann, 2015). Paired associative stimulation (PAS), which induces plasticity in the motor cortex, was reported to alter the subsequent learning effect of motor practice in a way that was dependent on the interval between PAS and motor learning (Jung and Ziemann, 2009). In addition, voluntary muscle contraction is known to influence the effects of theta burst stimulation (TBS) on brain plasticity, and the temporal relationship between muscle contraction and TBS (i.e., contraction before, during, or after TBS) may affect the direction of influence, as it can enhance, abolish, or reverse the effects of TBS (Gentner et al., 2008; Huang et al., 2008). These results suggest that the temporal relationship between cortical stimulation and PES could affect plasticity in both the spinal cord and brain.

Intermittent theta burst stimulation (iTBS) is a repetitive transcranial magnetic stimulation (rTMS) protocol that uses a short stimulation period (190 s). iTBS alters motor cortex excitability comparably to other rTMS and tDCS protocols that involve longer stimulation (10 or 20 min) (Huang et al., 2005; Jeffery et al., 2007; McAllister et al., 2009; Goldsworthy et al., 2012; Tatemoto et al., 2013), and is also capable of enhancing motor cortical excitability evoked by stimulation of the cortical lower limb area (Jeffery et al., 2007; Tatemoto et al., 2013; Giboin et al., 2016). These observations suggest that iTBS might be usable to modify PES-induced plasticity, and furthermore the brief stimulation used in this protocol makes it more suitable than other protocols for examining the influences of the timing and magnitude of motor cortical excitability changes on the spinal plasticity induced by PES.

In the present study we applied iTBS to modulate motor cortical excitability, and examined its time-dependent effects on the enhancement of spinal RI induced by PES in healthy individuals. In addition, we tested the effects of iTBS delivered before and after PES on motor cortical excitability by using TMS-elicited motor-evoked potentials (MEP) to elucidate the correlation between changes in motor cortical excitability and spinal plasticity.

## Materials and Methods

### Participants

Ten healthy subjects who responded positively to TBS in previous experiments [eight women and two men, aged 33–58 years {mean ± standard deviation (SD), 42.8 ± 8.3 years}] were recruited from our bank of volunteers. Previous investigations have demonstrated that healthy individuals show relatively consistent responses to repeated iTBS sessions (Hinder et al., 2014). None of the participants had a history of neurological disease or were receiving any medication affecting the central nervous system. All participants provided written informed consent prior to participation. The study was approved by the Institutional Review Board of Chang Gung Memorial Hospital in Taiwan and conformed to the tenets of the Declaration of Helsinki.

### Electromyography

The participants were seated in a comfortable chair with a backrest and a headrest. The angle of the hip joint in the sitting position was set such that it ranged from 70 to 80 degrees of flexion, the knee was set at 70–80 degrees of flexion, and the ankle was maintained at 10 degrees of plantar flexion using a rigid ankle brace. Electromyography (EMG) was performed using Ag/AgCl-plated surface electrodes (diameter 1 cm) placed 2 cm apart over the tested muscles in the right lower limb. EMG data were obtained from the soleus (SOL, for RI and TMS tests) and tibialis anterior (TA, for TMS tests) muscles. The EMG data were amplified and band-pass filtered (3 Hz to 2 kHz) using Digitimer D360 amplifiers (Digitimer Ltd., Welwyn Garden City, Hertfordshire, United Kingdom). Signals were recorded at a sampling rate of 5 kHz using a Power 1401 data acquisition interface (Cambridge Electronic Design Ltd., Cambridge, United Kingdom), and stored on the computer for subsequent analysis using Signal software (Cambridge Electronic Design Ltd., Cambridge, United Kingdom). The EMG activity was monitored online. If the muscle was not fully relaxed, the trial was rejected and performed over again.

### Intermittent Theta Burst Stimulation

The iTBS protocol consisted of 10 bursts, each composed of three stimuli at 50 Hz repeated at a theta frequency of 5 Hz every 10 s, for a total of 600 stimuli (190 s) (Huang et al., 2005). TMS was performed using a MagPro X100 stimulator [Medtronic and (currently) MagVenture A/S, Denmark] to deliver biphasic TMS pulses through a figure-eight coil with a diameter of 70 mm (MC-B70). The stimulating coil was placed over the leg area of the primary motor cortex that was optimal for eliciting responses in the right TA muscle, and oriented such that the current in the brain flowed in a posterior to anterior direction through this site. The stimulation intensity was 80% of the active motor threshold (AMT). The AMT was defined as the minimum stimulation intensity required to evoke a liminal motor potential in the TA (greater than 200 μV in 50% of the 10 trials) while inducing isometric contraction with EMG amplitudes of 100 μV in the TA. For the sham stimulation, iTBS was delivered at 60% of the AMT with the coil turned over to further reduce the current intensity on the leg area of the primary motor cortex, further reducing the intensity to the cortex (Huang et al., 2009).

### Patterned Electrical Stimulation

We applied electrical stimulation to the common peroneal nerve (CPN) on the right leg using a train of 10 100-Hz pulses (1 ms pulse width) every 2 s for 20 min, using an intensity that evoked a 100 μV M wave response in the TA at rest, without producing movement of the foot (Perez et al., 2003).

### Experimental Paradigm

**FIGURE 1:**
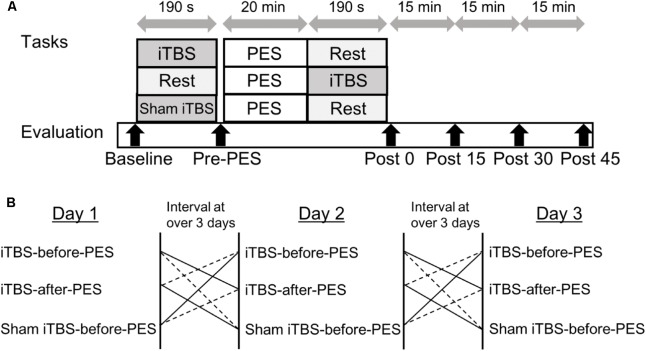
Experimental paradigm for intermittent theta burst stimulation (iTBS) before and after patterned electrical stimulation (PES). **(A)** Time course of the experiment. **(B)** Sequence of interventions.

The study employed a single-masked, sham-controlled, crossover design. All participants received iTBS and PES during three separate sessions on different days: (1) iTBS-before-PES, (2) iTBS-after-PES, and (3) sham iTBS-before-PES (**Figure 1**). A computer-generated list randomly assigned the order of the three sessions. The RI and motor cortex excitability (measured by the MEP amplitude) were assessed at baseline (before any stimulation); before PES (Pre-PES); and at 0 (Post0), 15 (Post15), 30 (Post30), and 45 min (Post45) after PES. At each time point, RI was assessed first, then motor cortex excitability. The three sessions were performed at the same time of day for each participant. The participants were instructed to avoid caffeine and any medication that may have affected the central nervous system before the tests. To prevent carry-over effects from previous interventions, washout intervals of 3 days or more were inserted between sessions.

### Reciprocal Inhibition

Reciprocal inhibition from the TA to SOL muscle was assessed using the SOL H-reflex conditioning-test paradigm. The H-reflex was elicited by stimulating the posterior tibial nerve at the popliteal fossa (1-ms rectangular pulse) with an anode on the patella. Throughout the experiment, the test H-reflex amplitude was maintained at 15–20% of the amplitude of the maximum motor response for the SOL muscle (Crone et al., 1990). Conditioned stimulation of the CPN was delivered using surface electrodes positioned below the fibular head. The intensity was calibrated to evoke a 100 μV response of the M wave in the TA at rest. The CPN-stimulating electrode was carefully positioned to avoid activation of the peroneus muscles, thus ensuring a more selective stimulation of the deep peroneal nerve. Electric pulses were supplied by two constant-current stimulators (DS7A; Digitimer, Welwyn, United Kingdom). To confirm optimal disynaptic RI, we checked the H-reflex at an inter-stimulus interval (ISI) of 0, 1, and 2 ms at the beginning of each session. The ISI for the conditioning test was set at 2, 20, or 100 ms to trigger inhibition through separate mechanisms (Mizuno et al., 1971). Inhibition at an ISI of 2 ms is called disynaptic RI (RI_2ms_) and is mediated by a spinal glycinergic disynaptic inhibitory pathway (Curtis, 1959; Tanaka, 1974). Inhibition at an ISI of 20 ms (RI_20ms_) is called short-latency presynaptic inhibition, which is thought to result from presynaptic Ia inhibition of afferent fibers that mediate the H-reflex (Mizuno et al., 1971). Inhibition at an ISI of 100 ms (RI_100ms_) is called long-latency presynaptic inhibition (Mizuno et al., 1971; Huang et al., 2009). The origin of this effect is less clear and may be attributed to presynaptic inhibition that is modulated through long-loop inhibitory connections beyond the spinal cord (Mizuno et al., 1971; Huang et al., 2009). Stimuli were administered every 8 s in a random order, for a total of eight trials per condition. The amount of RI (%) at each ISI was defined as: $\frac{\text{mean test H-reflex amplitude-mean conditioned H-reflex amplitude}}{\text{mean test H-reflex amplitude}}\times 100.$

### Motor Cortex Excitability

To assess changes in motor cortex excitability, we applied single-pulse TMS to the leg area of the primary motor cortex using a double-cone coil connected to a Magstim BiStim2 machine (Magstim Company, Whitland, United Kingdom). The hotspot of the primary motor cortex was confirmed based on induction of the largest MEP amplitude in the TA at rest. The stimulation intensity was adjusted to 120% of the resting motor threshold (RMT). The RMT was defined as the intensity at which 5 out of 10 stimuli generated a 100 μV MEP response in the TA at rest, and was measured at the beginning of each session to set the stimulus intensity for the TMS measurement. Ten stimuli were delivered every 5 ± 0.5 s at each time point (Cacchio et al., 2009).

### Statistical Analysis

The peak-to-peak amplitude of each H-reflex response or MEP was measured, and the mean amplitude was automatically calculated using custom-written software (NuCursor; Sobell Department, Institute of Neurology, University College, London, United Kingdom). Changes in the amount of RI were calculated by subtracting data obtained at each testing time point (Pre-PES, Post0, Post15, Post30, and Post45) from the baseline data. MEP amplitudes were normalized to the baseline amplitude (%) for statistical analysis. To confirm the relaxation of muscles, we calculated the root mean square (RMS) values of the background EMG activity over a 50 ms period before the stimuli for the H-reflex and TMS were delivered.

The Shapiro–Wilk test was used to determine whether the RI, MEP, RMT, and AMT data were normally distributed. To compare baseline data between protocols, we used one-way analysis of variance (ANOVA; for normally distributed data) or Kruskal–Wallis tests (for non-normally distributed data) on the non-normalized values of the RI, MEP, RMT, and AMT. A two-way repeated-measures ANOVA was used to assess the effects of the timing of iTBS on normally distributed data for each protocol (iTBS-before-PES, iTBS-after-PES, and sham iTBS-before-PES) and for each testing time (Pre-PES, Post0, Post15, Post30, and Post45). Paired *t*-tests with Bonferroni adjustments for multiple comparisons were performed for *post hoc* comparisons. For the data that were not normally distributed, Mann–Whitney *U*-tests with Bonferroni adjustments were performed to evaluate between-group differences. To investigate the relationship between changes in the amount of RI and changes in motor cortex excitability, we performed Pearson correlation analyses (for normally distributed data) or Spearman rank correlation analyses (for non-normally distributed data) between the amount of RI at Post0 and the MEP amplitude at Pre-PES. Results with *P*-values < 0.05 were considered statistically significant for all analyses. Statistical analyses were performed using IBM SPSS 23.0 (IBM Corp., New York, NY, United States) for Windows.

## Results

The Shapiro–Wilk test confirmed that all data except the normalized MEP values were normally distributed. Two-way repeated-measures ANOVAs revealed no significant interactions between the protocol and testing time and no main effect of the RMS of the background EMG on any outcome in any of the conditions.

### RI

At baseline, the mean ± SD of RI_2ms_ was 7.7 (2.6)% in the iTBS-before-PES session, 9.5 (3.9)% in the iTBS-after-PES session, and 10.3 (4.3)% in the sham iTBS-before-PES session. For RI_20ms_, the mean ± SD was 7.8 (5.3)% for iTBS-before-PES, 7.8 (4.3)% for iTBS-after-PES, and 9.7 (4.4)% for sham iTBS-before-PES. For RI_100ms_, the mean ± SD was 12.4 (8.1)% in the iTBS-before-PES session, 11.4 (7.4)% in the iTBS-after-PES session, and 16.3 (8.6)% in the sham iTBS-before-PES session. The baseline values of RI were not significantly different among the three protocols (ANOVA, RI_2ms_: *F*_2,27_ = 1.33, *P* = 0.281; RI_20ms_: *F*_2,27_ = 0.56, *P* = 0.579; and RI_100ms_: *F*_2,27_ = 1.02, *P* = 0.375). When comparing the three phases of RI at baseline, we found a significant main effect (*F*_2,87_ = 6.40, *P* = 0.003). *Post hoc* analyses revealed that RI_100ms_ was stronger than either RI_2ms_ (*P* = 0.017) or RI_20ms_ (*P* = 0.004).

**FIGURE 2:**
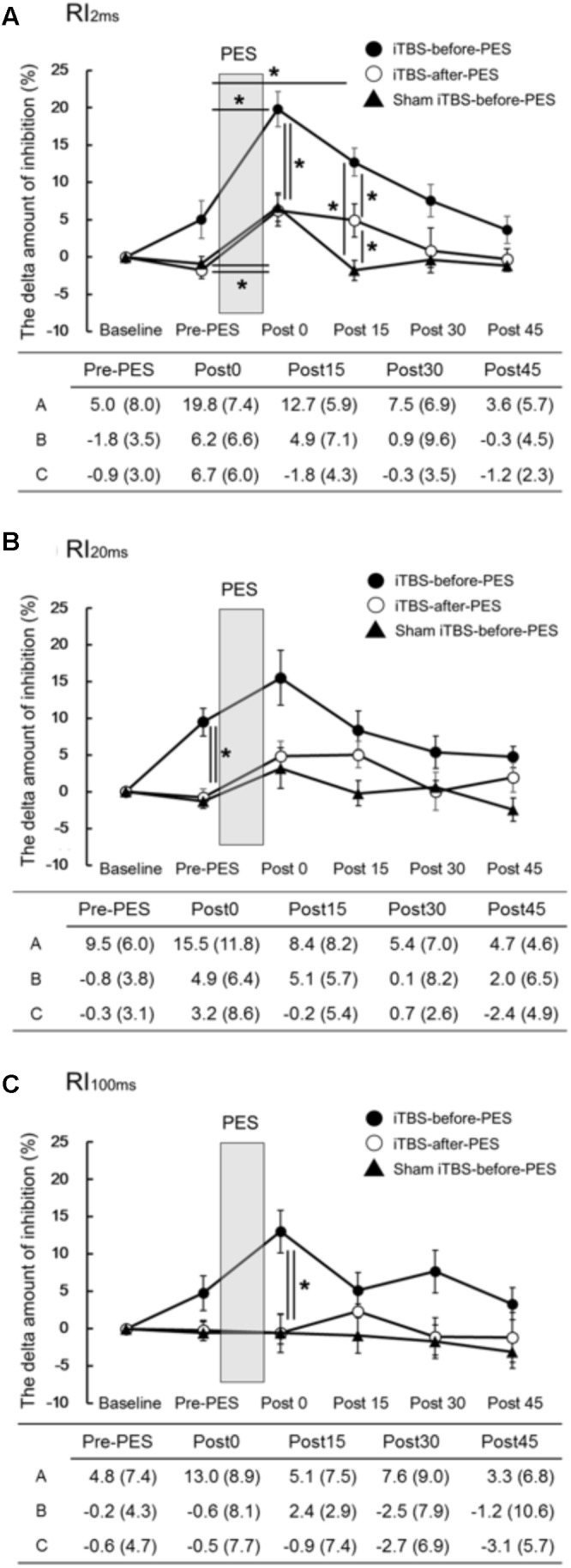
The effects of iTBS before and after PES on RI **(A–C)**. The values (%) of RI_2ms_ **(A)**, RI_20ms_ **(B)**, and RI_100ms_ **(C)** indicate the delta amount of inhibition, which was calculated by subtracting the values obtained at each testing time point from the baseline values. The values are presented as the mean ± standard error of the measurements in the figure and as the mean (SD) in the table. In the table, A shows iTBS-before-PES, B shows iTBS-after-PES, and C shows sham iTBS-before-PES. Asterisks indicate significant differences (*P* < 0.05) between Pre-PES and each intervention time point, or within the interventions.

The time course and values of the RI measures are shown in **Figure 2**. Significant interactions were found for RI_2ms_ (*F*_8,72_ = 2.79, *P* = 0.010, **Figure 2A**), RI_20ms_ (*F*_8,72_ = 2.35, *P* = 0.026, **Figure 2B**), and RI_100ms_ (*F*_8,72_ = 2.08, *P* = 0.049, **Figure 2C**). There were significant main effects of protocol and testing time on RI_2ms_ (protocol: *F*_1.20,10.82_ = 16.73, *P* < 0.001; testing time: *F*_4,36_ = 24.75, *P <* 0.001), RI_20ms_ (protocol: *F*_2,18_ = 8.10, *P* = 0.003; testing time: *F*_4,36_ = 6.47, *P* < 0.001), and RI_100ms_ (protocol: *F*_2,18_ = 12.45, *P* < 0.001; testing time: *F*_4,36_ = 2.11, *P* = 0.030).

Compared to the Pre-PES values, iTBS-before-PES significantly increased RI_2ms_ at Post0 (*P* = 0.002) and Post15 (*P* = 0.047), while iTBS-after-PES and sham iTBS-before-PES increased RI_2ms_ only at Post0 (*P* = 0.028 and 0.013, respectively). These results indicate that iTBS prior to PES enhanced PES-induced plasticity for 15 min or longer.

When the amount of RI was compared between protocols at each testing time point, priming iTBS was found to enhance the modulatory effect of PES on RI_2ms_ and RI_100ms_. RI_2ms_ was significantly increased by iTBS-before-PES (as compared to iTBS-after-PES and sham iTBS-before-PES) at the Post0 and Post15 time points (Post0: vs. iTBS-after-PES, *P* = 0.011; vs. sham iTBS-before-PES, *P* = 0.030. Post15: vs. iTBS-after-PES, *P* = 0.038; vs. sham iTBS-before-PES, *P* < 0.001). RI_100ms_ was enhanced only at the Post0 time point (vs. iTBS-after-PES, *P* = 0.004; vs. sham iTBS-before-PES, *P* = 0.007). On the other hand, iTBS-after-PES significantly increased RI_2ms_ when compared to sham iTBS-before-PES at the Post15 time point (*P* = 0.017).

Furthermore, iTBS-before-PES significantly increased the Pre-PES value of RI_20ms_ when compared to iTBS-after-PES (*P* = 0.006) and sham iTBS-before-PES (*P* = 0.002). This indicates that iTBS alone can enhance the inhibition measured by RI_20ms_.

### MEPs in the TA and SOL Muscles

The mean raw values (SD) of the MEP amplitude in the TA muscle at baseline were 0.27 (0.17) mV in the iTBS-before-PES session, 0.33 (0.24) mV in the iTBS-after-PES session, and 0.31 (0.20) mV in the sham iTBS-before-PES session. The baseline values of the MEP amplitude in the TA were not significantly different among the three protocols (Kruskal–Wallis test, *P* = 0.961). The mean raw values (SD) of the MEP amplitude in the SOL at baseline were 0.24 (0.11), 0.32 (0.22), and 0.30 (0.29) mV, and were not significantly different (Kruskal–Wallis test, *P* = 0.509). The mean values (SD) of RMT/AMT for the TA at baseline were 62 (10)%/54 (11)% of the maximum machine output in the iTBS-before-PES session, 62 (8)%/55 (12)% in the iTBS-after-PES session, and 63 (10)%/57 (10)% in the sham iTBS-before-PES session. There were no significant differences among the three protocols (RMT: ANOVA, *F*_2,27_ = 0.07, *P* = 0.930; AMT: ANOVA, *F*_2,27_ = 0.13, *P =* 0.875).

**FIGURE 3:**
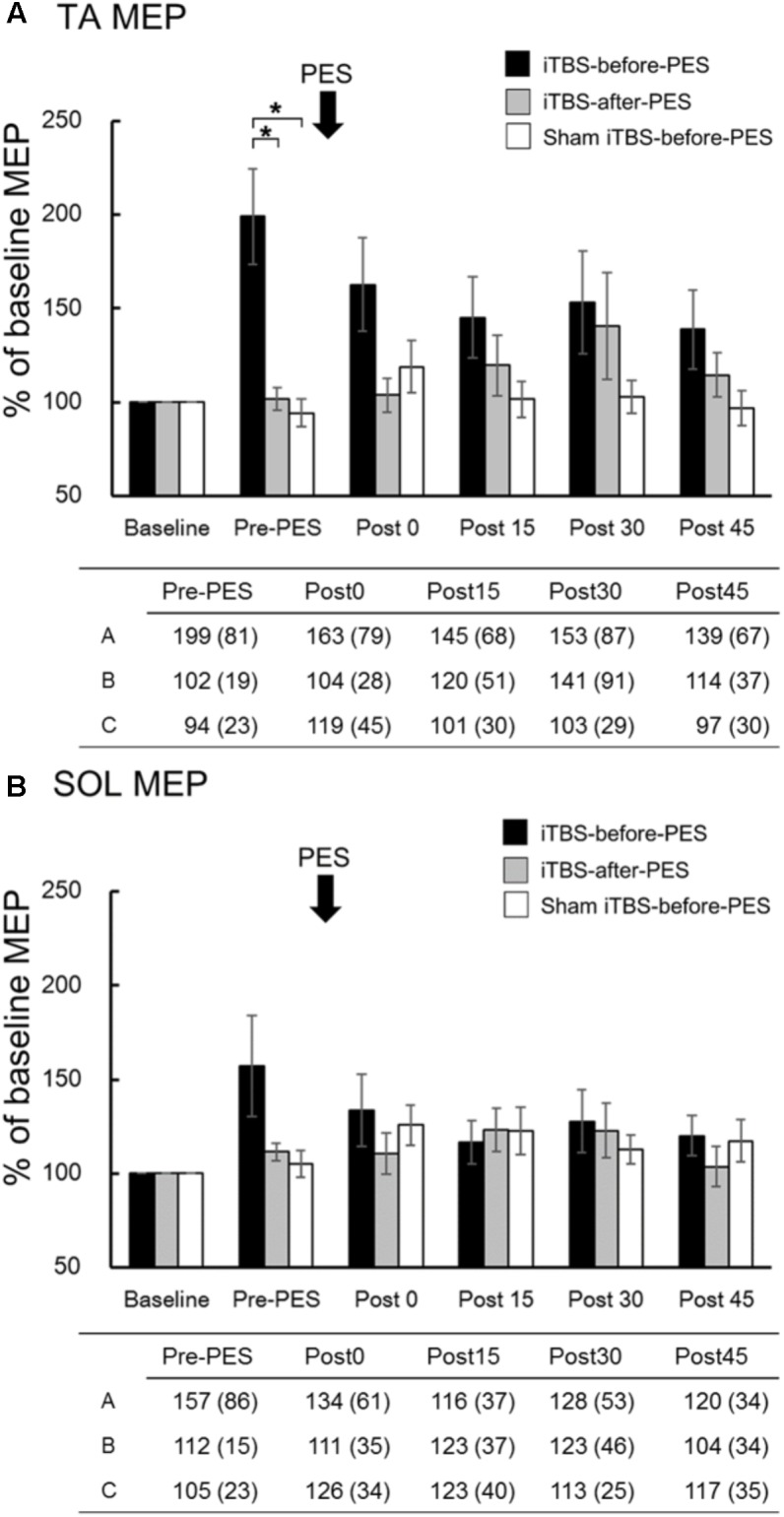
The effects of iTBS before and after PES on motor-evoked potentials (MEPs). MEP amplitudes at the tibialis anterior (TA; **A**) and soleus (SOL; **B**) muscles were normalized to the baseline amplitude (%) for each paradigm. The values (%) in the figures are presented as the mean ± standard error. The tables show the mean (±SD) for iTBS-before-PES **(A)**, iTBS-after-PES **(B)**, and sham iTBS-before-PES (C). Asterisks indicate significant differences (*P* < 0.05) among the interventions.

The time course and values of the MEP amplitude are shown in **Figure 3**. Since MEP amplitudes were not normally distributed, non-parametric tests were used to compare them. We used Mann–Whitney *U*-tests with Bonferroni correction to directly compare MEP amplitudes at each time point among protocols, based on the assumption that iTBS increases these amplitudes (Chung et al., 2016; Giboin et al., 2016). In addition, the Spearman rank correlation coefficient was used to analyze the relationship between RI at Post0 and the pre-PES of the MEP amplitude. We found that iTBS-before-PES significantly increased the Pre-PES of the MEP amplitude in the TA muscle compared to iTBS-after-PES (*P* = 0.004) and to sham iTBS-before-PES (*P* = 0.002; **Figure 3A**). iTBS-after-PES had no effects on the MEP amplitude at any testing timepoint compared with sham iTBS-before-PES. There were no significant differences among protocols regarding MEP amplitudes in the SOL muscle (**Figure 3B**).

### Correlation Between Changes in RIs and MEP Amplitude in the TA Muscle

**FIGURE 4:**
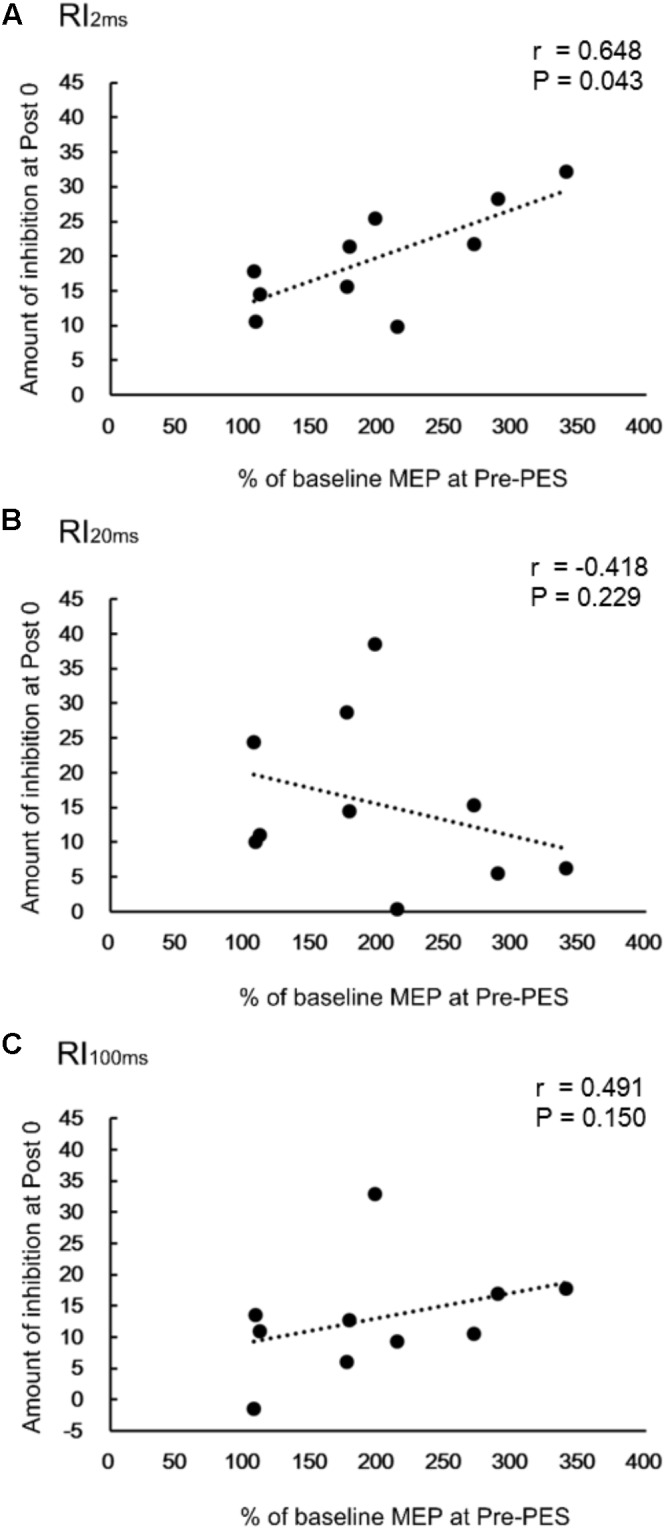
Correlations between change in reciprocal inhibition (RI) and MEPs. Correlation between percent changes in RI_2ms_ **(A)**, RI_20ms_ **(B)**, and RI_100ms_ **(C)** at Post0 time-point and the normalized MEPs in the TA muscle, for the iTBS-before-PES condition.

There was a significant positive correlation between RI_2ms_ at Post0 and the normalized amplitude of the TA MEP at the Pre-PES time point (*r* = 0.648, *P* = 0.043, **Figure 4A**) for iTBS-before-PES. However, no significant correlations were found between RI_20ms_ and the MEP amplitude in the TA (*r* = -0.418, *P* = 0.229, **Figure 4B**), or between RI_100ms_ and the MEP amplitude in the TA (*r* = 0.491, *P* = 0.150, **Figure 4C**). The results indicate that the enhancement of PES-induced spinal plasticity by iTBS was strongly related to the pre-state changes in cortical excitability modulated by iTBS.

## Discussion

Our results demonstrated that iTBS before, but not after, PES significantly increases and prolongs the PES-induced enhancement of disynaptic RI, and changes the effects of PES on long-loop presynaptic inhibition. The enhancement of disynaptic RI lasts for 15 min or longer. Our findings provide evidence that spinal plasticity, induced by peripheral sensory input via PES, can be enhanced by priming with iTBS. Moreover, iTBS-induced cortical excitability changes in the TA muscle before PES correlate with changes in disynaptic RI immediately after PES. This indicates that motor cortical excitability changes before PES plays an important role in the induction and maintenance of spinal plasticity.

### Priming Effects of iTBS on Spinal Plasticity Induced by PES

**FIGURE 5:**
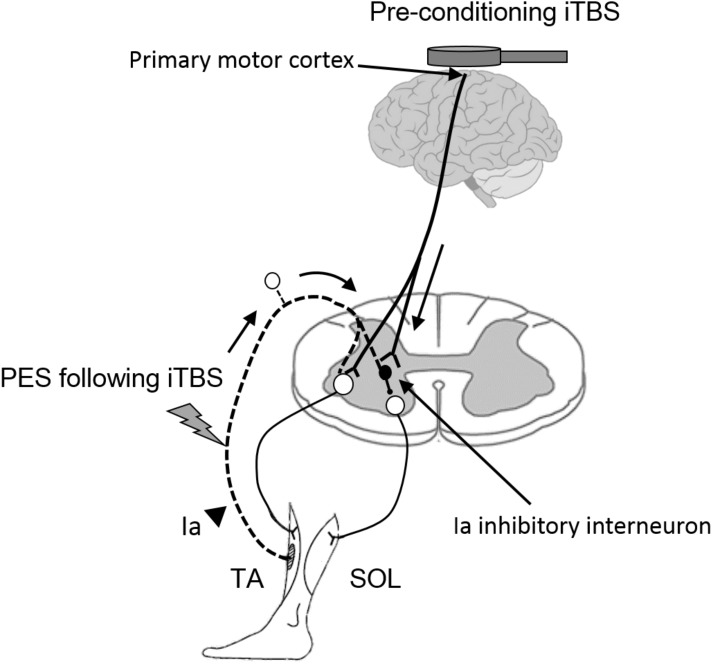
Hypothetical model for the modulatory effect of iTBS over the motor cortex on the subsequent induction of spinal plasticity induced by PES. White circles in the spinal cord denote spinal motor neurons. Black circles denote Ia inhibitory interneurons. Pre-conditioning by using iTBS changes cortical excitability and the state of Ia inhibitory interneurons, which receive convergent input from the motor cortex. The pre-state changes in the interneurons enhance the induction and maintenance of spinal plasticity induced by PES. TA, tibialis anterior; SOL, soleus.

Ia inhibitory interneurons, which project to SOL motor neurons, receive convergent inputs from the motor cortex and from the Ia afferents of the TA muscle (Nielsen et al., 1993; Masakado et al., 2001). iTBS is likely to modulate these types of corticospinal projections to spinal inhibitory circuits and thereby enhance the plasticity of disynaptic RI (**Figure 5**). This finding is supported by previous reports demonstrating that supraspinal modulation plays an important role in the induction and maintenance of spinal plasticity (Chen et al., 2006; Fujiwara et al., 2011; Yamaguchi et al., 2013, 2016; Takahashi et al., 2017).

Notably, we observed changes in the plasticity of long-latency presynaptic inhibition with iTBS-before-PES but not with the other two protocols. Accordingly, a previous study showed that the simultaneous application of anodal tDCS over the lower limb motor cortex and peripheral nerve electrical stimulation enhances long-latency presynaptic inhibition (Yamaguchi et al., 2016). However, the physiological mechanism underlying changes in long-latency presynaptic inhibition in these circumstances remains unclear. One possible explanation is that the combination of iTBS and PES modulates primary afferent depolarization (PAD) interneurons that receive convergent input from the motor cortex and from the Ia afferents of the TA muscle (Jankowska et al., 1981; Meunier and Pierrot-Deseilligny, 1998), and that these interneurons mediate long-latency presynaptic inhibition of Ia terminals, which project to SOL motor neurons (Mizuno et al., 1971; Iles and Pisini, 1992; Pierrot-Deseilligny and Burke, 2012). This finding suggests that the combination of cortical excitability changes and peripheral sensory inputs is a valid strategy to activate PAD interneurons and thereby induce plasticity in long-latency presynaptic inhibition.

The current results highlight the importance of the time sequence of cortical stimulation and sensory inputs for spinal plasticity. Changes in the plasticity of disynaptic RI were stronger for iTBS-before-PES than for iTBS-after-PES, and the effect on long-latency presynaptic inhibition was only seen when iTBS was given before PES. The enhancement of spinal plasticity by priming iTBS may be mediated by heterosynaptic metaplasticity, whereby iTBS-induced changes in corticospinal inputs modulate the subsequent PES-induced plasticity (Holland and Wagner, 1998; Abraham, 2008; Hulme et al., 2014; Parker, 2015). Many studies have reported interactions among the effects induced by different non-invasive brain stimulation protocols (Iyer et al., 2003; Ridding and Ziemann, 2010; Müller-Dahlhaus and Ziemann, 2015) through mechanisms of metaplasticity based on the Bienenstock–Cooper–Munro theory of bidirectional synaptic plasticity, which postulates that the threshold for induction of long-term potentiation or long-term depression is dynamically adjusted according to the history of activation in the synapse (Bienenstock et al., 1982). It is known that time is a critical factor affecting metaplastic interactions (Jung and Ziemann, 2009). The current results should promote our understanding of the importance of timing of cortical modulation on spinal plasticity induced by peripheral nerve electrical stimulation.

### Relationship Between PES-Induced Spinal Plasticity and Cortical Excitability

We found that the enhancement of spinal plasticity depended on primed cortical excitability, as altered by iTBS. The increased excitability following iTBS may in turn increase the descending volley from the motor cortex to spinal interneurons and enhance the synaptic strength (Pierrot-Deseilligny and Burke, 2012) of the disynaptic RI circuit. However, we found no correlation between changes in cortical excitability following iTBS and the enhancement of long-latency presynaptic inhibition after PES, nor in short-latency presynaptic inhibition. The simplest explanation for this result is that modulation from the motor cortex to the interneurons mediating short- and long-latency presynaptic inhibitions might be weak compared to the effects on the circuit responsible for disynaptic RI. Indeed, a study found that cortical inhibition evoked by transcranial cortical electrical stimulation clearly correlated with disynaptic RI, but not with short- and long-latency presynaptic inhibition (Iles and Pisini, 1992). Additionally, we showed that baseline inhibition activated by CPN stimulation is larger during long-latency presynaptic inhibition than during disynaptic RI. Previous studies have indicated that the range of long-latency presynaptic inhibition is approximately 20–40% under resting conditions in healthy individuals, while that of disynaptic RI is approximately 10–20% (Mizuno et al., 1971; Iles and Pisini, 1992; Fujiwara et al., 2011; Yamaguchi et al., 2013, 2016; Takahashi et al., 2017). Thus, there may be a ceiling effect in detecting the enhancement caused by increased cortical excitability in long-latency presynaptic inhibition.

### Effects of iTBS or iTBS Combined With PES on Motor Cortex Excitability and Spinal Inhibition

In agreement with previous studies on iTBS over the hand and leg area of the primary motor cortex (e.g., Huang et al., 2005; Chung et al., 2016; Giboin et al., 2016), we showed that iTBS facilitates corticospinal excitability of the TA muscle before PES, but does not significantly alter corticospinal excitability of the SOL muscle. The smaller degree of modulation in the SOL may be due to the fact that the intensity of iTBS was set to 80% of the AMT of the TA. In addition, a figure-eight coil is more focused and more likely to stimulate the TA muscle specifically (Thielscher and Kammer, 2004).

We did not observe a significant effect of iTBS on cortical excitability when we performed iTBS after PES. This may be a consequence of the afferent input stimulated by PES, which might cancel out the effects of the subsequent iTBS on motor cortex excitability. Indeed, previous studies have indicated that afferent stimulation from the CPN suppresses MEPs induced by single-pulse TMS (Roy and Gorassini, 2008; Zewdie et al., 2014). Similarly, the effects of TBS over the motor cortex of the upper limb are known to be modulated or even blocked by muscle activation that occurs around the time of TBS stimulation (Huang et al., 2008; Goldsworthy et al., 2012; Huang, 2016). Furthermore, Rambour et al. (2016) showed that application of iTBS after walking fails to increase motor cortex excitability in the TA muscle. On the other hand, Geertsen et al. (2011) reported that spinal interneuronal pathways modify the descending commands to spinal motor neurons and influence the MEP amplitudes elicited by TMS. Thus, the priming of afferent input stimulated by PES may change the state of synapses and synaptic transmitter release, thereby altering the response to TBS in the motor cortex or at the spinal level. This further confirms that the neuronal states of the brain and even of spinal networks are critical for obtaining optimal effects when using non-invasive brain stimulation (Huang et al., 2017).

### Comparison of the Effects of iTBS and tDCS on PES-Induced Plasticity and Spinal Inhibition

Compared to the 1-mA anodal tDCS used in a previous study (Yamaguchi et al., 2016), iTBS prior to PES may enhance PES-induced spinal plasticity in disynaptic RI and long-latency presynaptic inhibition over a slightly shorter duration (15 vs. 20 min). This is only speculative, as we did not measure the effects of iTBS for more than 20 min after the PES in the current study. Even though the 1-mA anodal tDCS may lead to longer-lasting enhancement than iTBS, the latter has some advantages, including better spatial and temporal resolution (Thielscher and Kammer, 2004; Huang et al., 2005; Jeffery et al., 2007; Bolognini et al., 2009; McAllister et al., 2009; Madhavan and Stinear, 2010; Roche et al., 2011; Goldsworthy et al., 2012; Tatemoto et al., 2013), which enabled us to study the effects of the temporal relationship of iTBS and PES.

In contrast to previous findings showing that anodal tDCS has no effect on short-latency presynaptic inhibition (Roche et al., 2011; Yamaguchi et al., 2016), we unexpectedly found that iTBS leads to its enhancement. The difference between tDCS and iTBS with respect to short-latency presynaptic inhibition suggests that different mechanisms underlie their after-effects (Di Lazzaro et al., 2010; Roche et al., 2015).

### Clinical Implications

Although the optimal timeframe needed for PES-induced spinal plasticity to improve functional recovery remains unclear, enhancement of spinal plasticity is of great importance for the improvement of motor function in patients with incomplete SCI (Bunday and Perez, 2012; Yamaguchi et al., 2016). PES, enhanced by iTBS, may be effective as an adjuvant therapy to other locomotor training therapies, such as treadmill walking with partial body weight support and robot-assisted locomotor training (Smith and Knikou, 2016), while its lasting effects may promote the functional recovery of patients with incomplete SCI.

### Limitations

The sample size of the current study was relatively small. Hence, some marginal results, e.g., the weak correlation between the enhancement of disynaptic RI and increased cortical excitability (*P* = 0.043), should be interpreted cautiously. Another limitation is that our study was conducted in healthy participants. A previous study found that iTBS over the primary motor cortex decreased (rather than increased) the size of MEPs in the arm muscles of patients with SCI (Fassett et al., 2017). Future studies are needed to test the current approach in patients with lower extremity paralysis.

## Conclusion

Priming with iTBS enhances and prolongs the modulatory effect of PES on spinal inhibitory circuits, indicating that pre-modulation of motor cortex excitability plays an important role in spinal plasticity. In contrast, PES prior to iTBS reduces the effect of iTBS on the primary motor cortex, suggesting that afferent inputs may modulate subsequent motor cortex plasticity or the output of motor plasticity at the spinal level. The current findings provide further insight into our understanding of the relationship between the timing of corticospinal excitability modulation and spinal RI in humans. Further studies are warranted to clarify the clinical application of non-invasive neuromodulation in patients with lower limb paralysis.

## Author Contributions

TY, TF, and Y-ZH provided the concept, research design, and project management. TY, TF, ML, and Y-ZH wrote the manuscript and procured funding. TY, S-CL, and Y-ZH recruited participants and collected data. TY, S-CL, YT, and KH performed the data analysis. Y-ZH provided facilities and equipment. TY, TF, KH, ML, and Y-ZH provided consultation (including review of manuscript before submission).

## Conflict of Interest Statement

The authors declare that the research was conducted in the absence of any commercial or financial relationships that could be construed as a potential conflict of interest.
